# What is the impact of the COVID-19 pandemic on emergency medicine residency training: an observational study

**DOI:** 10.1186/s12909-020-02267-2

**Published:** 2020-10-07

**Authors:** Hsiang-Yun Lo, Shen-Che Lin, Chung-Hsien Chaou, Yu-Che Chang, Chip-Jin Ng, Shou-Yen Chen

**Affiliations:** 1 Department of Emergency Medicine, Chang Gung Memorial Hospital and Chang Gung University College of Medicine, No. 5, Fuxing St., Guishan Dist., Taoyuan City, 333 Taiwan, Republic of China; 2 The Institute of Health Policy and Management, National Taiwan University, No. 17, Xu-Zhou Road, Taipei, 100 Taiwan; 3 Graduate Institute of Clinical Medical Sciences; Division of Medical Education, College of Medicine, Chang Gung University, No.259, Wenhua 1st Rd.,Guishan Dist, Taoyuan City, 333 Taiwan

**Keywords:** Emergency medicine, Education, Residents, COVID-19

## Abstract

**Background:**

The coronavirus disease 2019 (COVID-19) pandemic has engendered difficulties for health systems globally; however, the effect of the pandemic on emergency medicine (EM) residency training programs is unknown. The pandemic has caused reduced volumes of emergency department (ED) patients, except for those with COVID-19 infections, and this may reduce the case exposure of EM residents. The primary objective of this study was to compare the clinical exposure of EM residents between the prepandemic and pandemic periods.

**Methods:**

This was a retrospective study of EM resident physicians’ training in a tertiary teaching hospital with two branch regional hospitals in Taiwan. We retrieved data regarding patients seen by EM residents in the ED between September 1, 2019, and April 30, 2020. The first confirmed COVID-19 case in Taiwan was reported on January 11, so the pandemic period in our study was defined as spanning from February 1, 2020, to April 30, 2020. The number and characteristics of patients seen by residents were recorded. We compared the data between the prepandemic and pandemic periods.

**Results:**

The mean number of patients per hour (PPH) seen by EM residents in the adult ED decreased in all three hospitals during the pandemic. The average PPH of critical area of medical ED was 1.68 in the pre-epidemic period and decreased to 1.33 in the epidemic period (*p* value < 0.001). The average number of patients managed by residents decreased from 1.24 to 0.82 in the trauma ED (*p* value = 0.01) and 1.56 to 0.51 in the pediatric ED (*p* value = 0.003) during the pandemic, respectively. The severity of patient illness did not change significantly between the periods.

**Conclusions:**

The COVID-19 pandemic engendered a reduced ED volume and decreased EM residents’ clinical exposure. All portion of EM residency training were affected by the pandemic, with pediatric EM being the most affected. The patient volume reduction may persist and in turn reduce patients’ case exposure until the pandemic subsides. Adjustment of the training programs may be necessary and ancillary methods of learning should be used to ensure adequate EM residency training.

## Background

Coronavirus disease 2019 (COVID-19) is a novel viral disease that has spread rapidly and become a global pandemic. Health care systems worldwide have struggled to cope with the pandemic and prepare for a surge of patients with respiratory tract infections. In Taiwan, the emergency department (ED) patient volume has dropped with the progression of the pandemic. Similar situations have been noted in several countries in Europe and some states in the United States [1–5]. This drop can be attributed to people’s fear of being infected and being a burden on the health care system, as reported in multiple countries [5]. Additionally, people staying at home due to the quarantine policy of government or fear of COVID-19 infection may lower the traffic accidents, non-COVID infectious diseases, sports injuries, etc. This also caused decreased ED volume.

Studies have reported the effect of the COVID-19 pandemic on residency training programs in urology, surgery, and ophthalmology [6–9]. The rapid decrease in clinical and surgical activities has posed challenges to residency training programs in several specialties. The pandemic has also affected the mental health of residents, which may further interfere with their clinical learning [10, 11]. Emergency physicians have been at the frontline of the pandemic; nevertheless, according to our review of the relevant literature, studies have yet to be conducted on the effect of the pandemic on emergency medicine (EM) residency training. The reduced ED volume caused by the pandemic may affect EM residents’ clinical case exposure, thus engendering insufficient clinical experience. The extent of case exposure reduction is unknown. To address this knowledge gap, the present study explored the effect of reduced ED volume on EM residency training; the findings may provide suggestions to EM educators regarding the change in the learning conditions of EM residents during the pandemic.

## Methods

### Study design

This was a retrospective study of EM resident physicians’ training during the COVID-19 pandemic. To evaluate the effect of the COVID-19 pandemic on EM residency training programs, we divided the study period into two periods: prepandemic and pandemic periods. The first confirmed COVID-19 case in Taiwan was reported on January 11, 2020, and the larger outbreak began in February 2020. The EM training rotation is structured by month; therefore, the pandemic period in our study was defined as spanning from February 1, 2020, to April 30, 2020. The EM training year began on September 1, 2019; hence, the prepandemic data were considered as those obtained between September 1, 2019, and January 31, 2020. The study was approved by our institutional review board (IRB no. 202000945B0).

### Study setting and population

The study was conducted at a university-affiliated tertiary teaching hospital with a 3600-bed capacity and an estimated annual ED volume of 180,000 patient visits. Two branch hospitals with an estimated ED volume of 48,000 and 78,000 patient visits were also included. The three hospitals are located in three different cities in northern Taiwan.

The ED has 63 board-certified EM faculty members and conducts an EM training program for 7–10 resident physicians annually. The training residents rotate among the three hospitals—namely one general hospital in Linkou and two branch hospitals in Taipei and Keelung—on a monthly basis. The general hospital in Linkou is a tertiary medical center, and the branch in Keelung and that in Taipei are regional hospitals. Table 1 presents the patient groups seen by residents enrolled in different rotations. The general hospital has an adult ED and a pediatric ED. The adult ED includes a medical ED and trauma ED. The medical ED has care facilities for patients with triage acuity levels 1 and 2 and facilities for patients who require critical care. In the general hospital, residents see new patients in the medical ED critical care area, trauma ED, and pediatric ED according to their EM rotations. In branch hospitals, EM residents see adult patients with trauma or nontrauma conditions. At the time of study, the EM residency program involved 3.5 years of training, including 2-month trauma ED and 2-month pediatric ED rotations, in the general hospital, according to the criteria stipulated by the Taiwan Society of Emergency Medicine.

**Table 1:** EM residents’ training courses

Linkou general hospital	
Critical areas of medical ED	Nontraumatic adult patients with triage acuity levels 1 and 2, or patients requiring critical care facilities
Trauma ED	Traumatic patients
Pediatric ED	Nontraumatic pediatric patients (age < 18 years)
Taipei branch, adult ED	Adult patients (including nontrauma and trauma cases)
Keelung branch, adult ED	Adult patients (including nontrauma and trauma cases)

The study included data from September 1, 2019, to April 30, 2020. The electronic medical record system was queried, and a database with the following information was generated: patient gender, patient age, patient triage acuity level, the first physician seen, and disposition. Only the data of patients who were first seen by residents were included in the study. Resident shift schedules were reviewed, and working hours were calculated.

### Outcome measurements

Data on new patients seen by residents during the prepandemic period were compared with those of patients seen by residents during the pandemic period. The main outcome was the average number of patients seen by residents per hour during the prepandemic and pandemic periods. The demographic characteristics and triage acuity levels of patients seen by residents were also compared. Data on different training courses were analyzed separately.

### Statistical analysis

Data were analyzed using SPSS software (version 13.0 for Windows; SPSS Inc., Chicago, IL, USA). Regarding descriptive statistics, categorical variables are presented as numbers and percentages. The recorded patient visits during the prepandemic and pandemic periods were compared using the Mann–Whitney U test for continuous variables and Pearson’s chi-square or Fisher’s exact tests for categorical variables. A *p* value of < 0.05 was considered statistically significant.

## Results

**Fig. 1:**
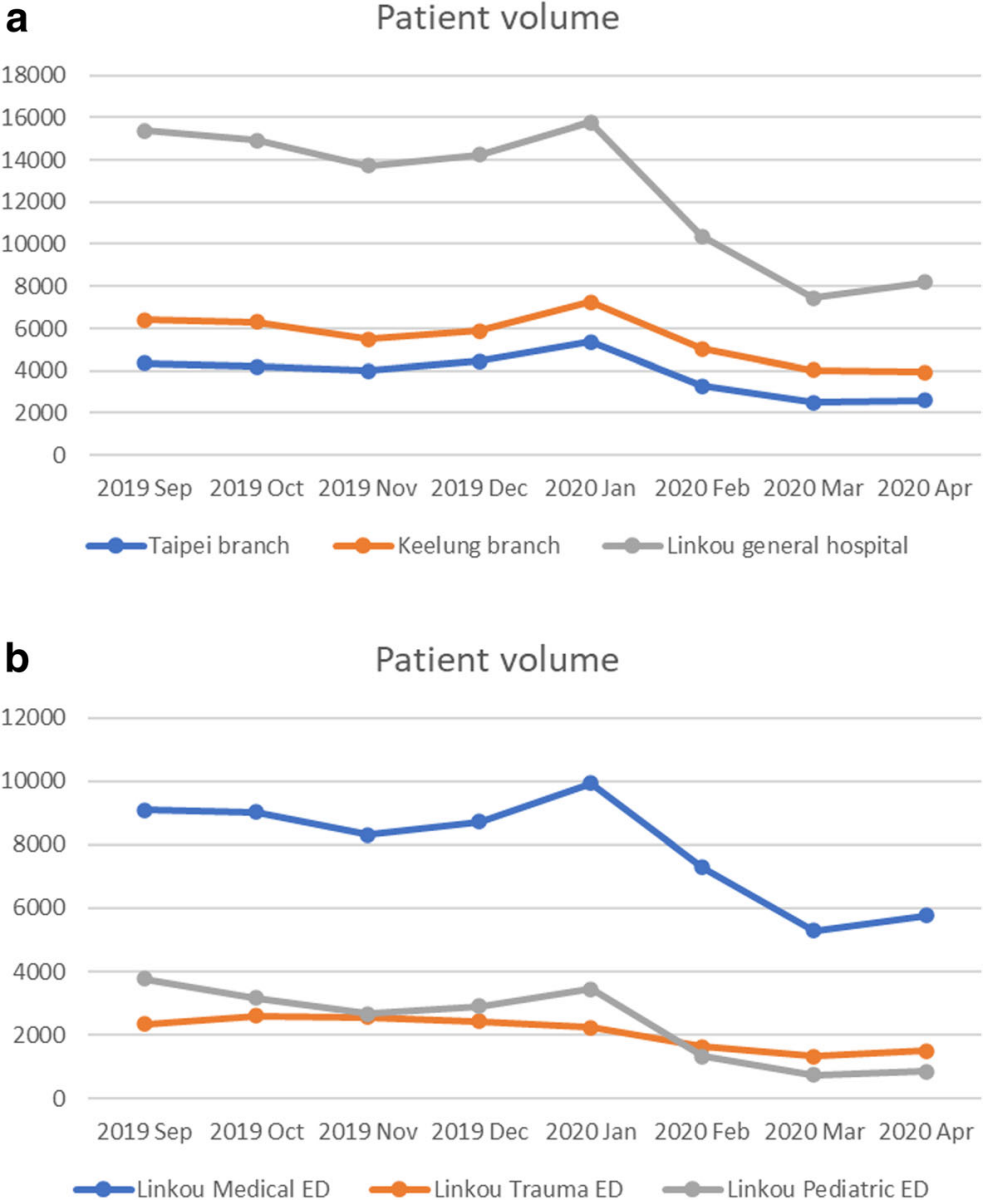
**a** ED visits to the Linkou general hospital and the Taipei and Keelung branches. **b** ED visits in different ED departments of the Linkou general hospital

The data collected from the three hospitals for the 8-month study period comprised more than 175,000 ED visits. A total of 51,337 patients were managed by 36 EM residents during the 8-month study period. Figure 1 presents the number of ED visits in each month. Figure 1a shows the patient volumes of the three hospitals. The ED volumes of the three hospitals exhibited similar trends, namely decreased volume since February 2020. We observed that the average patient volume per month decreased by more than 30% in all three hospitals during the pandemic period. The volumes of different EDs at the general hospital are presented in Fig. 1b; similarly, we noted a reduction in patient volumes. The patient volumes of the medical ED, trauma ED, and pediatric ED decreased clearly during the pandemic period. The patient volume during the pandemic period was nearly half and one-third of that during the prepandemic period in the trauma ED and pediatric ED, respectively.

Z

In the adult EDs of the Taipei and Keelung branches, the mean number of patients seen by residents during the pandemic period decreased relative to that during the prepandemic period (Table 2). The patients per hour (PPH) seen by residents in the Taipei and Keelung branches differed significantly between the prepandemic and pandemic periods. The average PPH of Taipei branch was 2.32 in the pre-epidemic period and decreased to 1.77 in the epidemic period (*p* = 0.006). In the Keelung branch, the average PPH was 2.30 in the pre-epidemic period and dropped to 1.89 in the epidemic period (*p* < 0.001). We observed similar analysis results for the general hospital. The average PPH seen by residents in rotations in the medical ED critical care area (from 1.68 to 1.33, *p* < 0.001), trauma ED (from 1.24 to 0.82, *p* = 0.01), and pediatric ED (from 1.56 to 051, *p* = 0.003) during the pandemic period were significantly lower than those recorded during the prepandemic period.

**Table 2:** Patients per hour seen by residents

	Pre-epidemic period					Epidemic period			
Time	2019/09	2019/10	2019/11	2019/12	2020/01	2020/02	2020/03	2020/04	*p* value
Taipei branch	1.72	2.53	2.41	2.35	2.61	2.28	1.44	1.58	*p* = 0.006
Keelung branch	2.43	2.26	2.29	2.00	2.53	2.19	1.73	1.75	*p* < 0.001
Linkou general hospital									
Critical area of Medical ED	1.58	1.63	1.76	1.87	1.55	1.52	1.23	1.23	*p* < 0.001
Trauma ED	^a^	1.17	0.92	1.17	1.17	0.86	0.73	0.88	*p* = 0.01
Pediatric ED	1.64	1.54	1.23	1.64	1.73	0.73	0.35	0.46	*p* = 0.003

^a^no EM resident rotated to trauma ED on 2019/09

The demographic characteristics of patients seen by residents in the Taipei and Keelung branches were analyzed. In Taipei branch, the percentage of patients with triage acuity levels 1 and 2 was higher during the pandemic period (4.88%) than it was during the prepandemic period (3.92%) (Table 3). A similar result was noted in Keelung branch, which the percentage of acuity 1 and 2 were 9.8 and 10.4% in pre-epidemic period and the epidemic period respectively. The admission rate during the pandemic period relative to that during the prepandemic period was substantially higher in the Keelung branch (from 22.86 to 26.21%) but only slightly higher (from 6.95 to 7.14%) in the Taipei branch. In the general hospital, the percentage of patients with triage acuity levels 1 and 2 did not differ between the two periods in the medical ED critical care area, but the admission rate increased from 56.84 to 61.31% (*p* < 0.001) during the pandemic period (Table 4). Similar results were obtained for such patients in the pediatric ED, which admission rate increased from 19.63 to 24.15% (*p* = 0.04). In the trauma ED, no significant difference was noted in the percentage of patients with either triage acuity level or the admission rate between the two periods.

**Table 3:** Characteristics of patients seen by residents in Taipei and Keelung branches

	Pre-epidemic period	Epidemic period	*P* value
Taipei branch			
Average patient number per month	2034.40	1371.67	
Gender, Male	4531 (44.5%)	1932 (47.0%)	*p* = 0.009
Age, years	50.34 ± 22.04	50.34 ± 22.20	*p* = 0.811
Triage level			*p* = 0.026
Level 1	87 (0.86%)	45 (1.09%)	
Level 2	312 (3.06%)	156 (3.79%)	
Level 3	6399 (62.91%)	2610 (63.43%)	
Level 4	3110 (30.57%)	1224 (29.74%)	
Level 5	263 (2.59%)	80 (1.94%)	
Admission	707 (6.95%)	294 (7.14%)	*p* = 0.681
Keelung branch			
Average patient number per month	2773.40	2233.33	
Gender, Male	6875 (49.45%)	3419 (51.03%)	*p* = 0.034
Age, years	54.97 ± 21.64	54.38 ± 21.61	*p* = 0.049
Triage level			*p* < 0.001
Level 1	363 (2.62%)	236 (3.52%)	
Level 2	995 (7.18%)	461 (6.88%)	
Level 3	10,348 (74.62%)	4727 (70.55%)	
Level 4	2084 (15.03%)	1231 (18.37%)	
Level 5	76 (0.55%)	44 (0.66%)	
Admission	3170 (22.86%)	1756 (26.21%)	*p* < 0.001

**Table 4:** Characteristics of patients seen by residents in Linkou general hospital

	Pre-epidemic period	Epidemic period	*P* value
Critical area			
Average patient number per month	1810.00	1546.67	
Gender, Male	4994 (55.18%)	2630 (56.68%)	*p* = 0.055
Age, years	64.00 ± 19.64	62.52 ± 19.48	*p* < 0.001
Triage level			*p* = 0.166
Level 1	1120 (12.38%)	601 (12.95%)	
Level 2	3744 (41.37%)	1853 (39.94%)	
Level 3	6399 (45.27%)	2125 (45.80%)	
Level 4	87 (0.96%)	58 (1.25%)	
Level 5	2 (0.02%)	80 (1.94%)	
Admission	5144 (56.84%)	2845 (61.31%)	*p* < 0.001
Trauma area			
Average patient number per month	300.80	291.67	
Gender, Male	873 (58.05%)	535 (61.14%)	*p* = 0.138
Age, years	38.83 ± 23.69	40.30 ± 22.42	*p* = 0.067
Triage level			*p* = 0.082
Level 1	8 (0.53%)	14 (1.60%)	
Level 2	208 (13.83%)	107 (12.23%)	
Level 3	1161 (77.19%)	674 (70.03%)	
Level 4	123 (8.18%)	77 (8.8%)	
Level 5	4 (0.27%)	3 (0.34%)	
Admission	286 (19.02%)	181 (20.69%)	*p* = 0.323
Pediatric area			
Average patient number per month	356.60	138.00	
Gender, Male	1008 (56.53%)	234 (56.76%)	*p* = 0.932
Age, years	4.93 ± 4.34	6.02 ± 5.42	*p* = 0.024
Triage level			*p* = 0.304
Level 1	252 (14.13%)	48 (11.59%)	
Level 2	351 (19.69%)	92 (22.22%)	
Level 3	1103 (61.86%)	251 (60.63%)	
Level 4	76 (4.26%)	22 (5.31%)	
Level 5	1 (0.06%)	1 (0.24%)	
Admission	350 (19.63%)	100 (24.15%)	*p* = 0.040

## Discussion

The objective of EM residency training is to enhance residents’ knowledge and breadth of clinical experience. Specifically, the primary goal of EM residency training programs is to ensure that residents gain the required clinical experience to master the knowledge and clinical skills necessary to practice EM [12]. Case exposure based on seeing a variety of patients with varying acuity levels, chief complaints, and diagnoses is crucial for the development of comprehensive experience [13–15]. Our study demonstrated that the decrease in ED volume caused by the COVID-19 pandemic significantly affected the case exposure of EM residents. Inadequate clinical exposure may hinder residents in attaining clinical competency and experiencing core EM diagnoses [16]. The adequate patient volume recommended for EM residency training is unknown; nevertheless, previous studies have reported that 15,000 annual ED visits to a training hospital and 1.1–1.4 PPH on average for trainees are reasonable [14, 17, 18]. The PPH for residents in the adult ED decreased significantly during the pandemic period relative to that during the prepandemic period. However, if the pandemic persists, the long-term effect of the change in patient volume on resident training is unknown. Although the ED volume was lower during the pandemic period, the severity of illness in patients visiting the ED did not increase significantly as expected. Training in the management of critically ill patients is essential for emergency physicians, and the training would be affected by the number of patients seen during the pandemic period [19]. Accordingly, a monitoring system for assessing residents’ case exposure regularly is necessary during the COVID-19 pandemic [13].

Our study revealed that the pediatric patient volume was most affected during the pandemic period. The volume of patients seen by residents during the pandemic period was less than half of that observed during the prepandemic period. The acuity level of patients remained unchanged during the pandemic period. Fear of having COVID-19 or getting infected by the virus in the hospital could be a reason for parents not bringing their children to the hospitals. The importance of pediatric training in EM residency programs has been mentioned in previous reports, but the sufficiency level of training is inconclusive [16, 20, 21]. Previous surveys have revealed that EM faculties perceived that they were less prepared to manage pediatric patients than they were for managing adult patients [22]. Decreased pediatric case exposure in EM training during the pandemic period could exacerbate the issue of insufficient pediatric training of EM residents. Revising the EM training course to increase pediatric training time should be considered if the reduction in pediatric patient volume persists; however, such an adjustment may affect other portions of EM residency training programs. Ancillary methods, such as web-based learning and high-fidelity simulation, may help toward pediatric EM training during the pandemic period [23–26].

The volume of trauma patients also dropped during the pandemic period in our study. Possible reasons for this drop are people’s reduced frequency of leaving the house because of fear of COVID-19 and quarantining of high-risk groups. A decrease in trauma patient volume reduces the number of emergency procedures performed by residents and reduces residents’ experience in managing trauma patients, which can lead to poor educational outcomes of traumatology training [27]. Although the magnitude of decrease in trauma patient volume was not as high as that observed for the volume of pediatric patients, close observation and evaluation by senior doctors are necessary to ensure that traumatology training is sufficient for EM residents.

At the time of writing, the COVID-19 pandemic continues to be a global health crisis. Reduced ED volumes are continuing to be noted in hospitals worldwide. Our study may serve to remind EM educators to evaluate their resident training programs during the pandemic. Along with addressing the pandemic and handling the surge of respiratory tract infection cases, regular EM residency training with other EM core diagnoses should be considered. Some study had proposed several innovative solutions may help sustain training for other subspecialty during the pandemic [8]. It included the flipped classroom model, online practice questions, teleconferencing in place of in-person lectures, involving residents in telemedicine clinics, procedural simulation, and the facilitated use of videos. These methods may be used to maintain EM residency training. Adjusting training programs and adding ancillary training methods may be necessary to ensure adequate training for EM residents.

### Limitations

This study has several limitations. First, because our study was retrospective and was limited to three hospitals in a single country; hence, selection bias may exist and it may not be representative to situations in other hospitals worldwide. The effect of the COVID-19 pandemic may vary in different countries; additional data from other coun7tries are thus required to further evaluate EM education during the COVID-19 pandemic in other countries. Second, A so-called “wash-out period”, which indicates the transition period from pre-epidemic to epidemic condition, was not reflected in our study. Although the effect of wash-out period existed, it was difficult to define the actual date of this period and included into the study. Third, seasonal and temporal factors which may affect ED volume were not included in our study. These factors may cause some differences. Finally, our study mainly focused on the role of clinical exposure in EM residency training. For clinical exposure, the case diversity was also important. Our data did not include patient presentation, diagnosis, and prognosis, and comparison of these factors was not available and the effect was unknown. Although case exposure cannot be replaced by other ancillary methods, it is not the only measure of EM residency training., our study did not assess educational outcomes between the prepandemic and pandemic periods and did not investigate the effect of the reduced level of case exposure during the pandemic on residents’ learning. Further research is necessary to compare the two periods with respect to other indicators to derive a complete assessment of residents’ clinical training and performance.

## Conclusions

The COVID-19 pandemic engendered reduced ED volume, thus reducing the clinical exposure of EM residents during their training. All portions of EM training were affected by the pandemic; the effect of the pandemic on pediatric EM was the most severe because of a profound decrease in pediatric patient volume. If the COVID-19 pandemic persists, its effect on residents’ training could lead to poorer EM educational outcomes compared with the outcomes observed during the prepandemic period. The learning conditions of EM residents must be regularly monitored and assessed. Adjustments to EM residency training programs may be necessary, and ancillary methods of EM learning would be helpful in the scenario of a prolonged pandemic.

## Data Availability

The dataset supporting the conclusions of this article is included within the article. The data in this study are available from the corresponding author upon reasonable request.

## Abbreviations

EM: Emergency medicine
ED: Emergency department
COVID-19: Coronavirus disease 2019
PPH: Patients per hour
